# Effectiveness of a telephone-delivered psycho-behavioural intervention on depression in elderly with chronic heart failure: rationale and design of a randomized controlled trial

**DOI:** 10.1186/s12888-019-2135-2

**Published:** 2019-05-27

**Authors:** Ying Zhang, Xiaozhen Lv, Wei Jiang, Yun Zhu, Weixian Xu, Yongdong Hu, Wanxin Ma, Peiyun Sun, Qingling Yang, Yulan Liang, Feng Ren, Xin Yu, Huali Wang

**Affiliations:** 1 Peking University Institute of Mental Health (Sixth Hospital), Beijing, China; 2 National Clinical Research Center for Mental Disorders & NHC Key Laboratory of Mental Health, Peking University Sixth Hospital, Beijing, China; 3 Beijing Dementia Key Laboratory, Beijing, China; 4 Department of Psychiatry, Duke University Medical Center, Durham, NC USA; 5 Peking University Third Hospital, Beijing, China; 6 Beijing Chaoyang Hospital, Capital Medical University, Beijing, China; 7 Beijing Chaoyang Mental Health Center, Beijing, China; 8 Tieying Hospital of Fengtai District, Beijing, China; 9 Tiancun Community Health Center, Haidian District, Beijing, China; 10 Beijing Anzhen Hospital, Capital Medical University, Beijing, China; 11 Peking University Shougang Hospital, Beijing, China

**Keywords:** Depression, Chronic heart failure, Telephone-delivered, Psycho-behavioural intervention, Elderly

## Abstract

**Background:**

Depression is common among chronic heart failure (CHF) patients, and it is associated with significant re-hospitalization and mortality as well as lower quality of life. While psychotherapy is efficacious treatment for depression, the effect for depression among CHF is uncertain. And barriers preclude widely utility of psychotherapy among the elderly. Telephone-delivered psycho-behavioural intervention specifically focuses on depression among the elderly with CHF, and could be a promising alternative to conventional treatment. The present study was designed to prospectively investigate the effect of a telephone-delivered psycho-behavioural intervention on depression in the elderly with chronic heart failure (CHF).

**Method/design:**

In this prospective, multicentre, parallel, randomized, and controlled trial, 236 participants with depression associated with CHF (New York Heart Association classes II and III) will be enrolled. The study will consist of a 12-week intensive intervention and a 24-week maintenance intervention. Eligible participants will be randomized to either the intervention arm or the control arm. During the intensive phase, participants will receive either a guided telephone psycho-behavioural intervention or regular telephone contacts from the counsellors weekly. During the maintenance phase, participants will receive either psychological behavioural support or regular telephone contacts monthly from counsellors. Depressive symptoms, cardiac outcome and quality of life will be assessed at baseline and weeks 1, 2, 4, 8, 12, 24 and 36. Participants will undergo echocardiography and the plasma concentrations of N-terminal pro-brain natriuretic peptide (NT-proBNP) tested at baseline, weeks 12 and 36. The primary outcome is the response rate of depression, from baseline to week 12. The second outcomes include the change in cardiac function, quality of life and severity of depressive symptoms during the trial.

**Discussion:**

To our knowledge, this study is the first prospective randomized trial to test the effective of the telephone-delivered psycho-behavioural intervention on depression in the elderly with CHF. The findings are expected to provide a new and evidence-based approach for depression among the elderly with CHF.

**Trial registration:**

The trial was registered at www.clinicaltrials.gov (identification number: NCT03233451) on 28 July 2017 and updated on 18 August 2017.

## Background

The prevalence of depression is approximately 21.5% among chronic heart failure (CHF) patients [1]. Previous studies found significant associations of depression with increased re-hospitalization and mortality of CHF as well as lower quality of life [2, 3]. Therefore, managing depression in CHF is critically important in clinical practice. It was reported that the risk of death significantly reduced when depression in patients with heart failure improved [4]. Depression may be a modifiable factor for improving the prognosis of CHF patients.

The effect of antidepressants on depression appears uncertain in the elderly CHF patients [5, 6]. And the use of antidepressants may be complicated by medical comorbidities, drug-drug interactions, and susceptibility of side effects in the elderly CHF. Psychological interventions may be a promising approach to the clinical management of depression among those CHF patients with depression.

Compared with regular telephone contacts, cognitive behavioural therapy (CBT) may improve depression in patients with CHF [7], both initially after CBT sessions and 3 months afterwards [8]. The secondary analysis of SADHART-CHF (Safety and Efficacy of Sertraline for Depression in Patients with CHF) study found that nurse-facilitated support may contribute to the improvements in depression scores in patients with CHF [9]. Previous research shows that tailored educational supportive care programme decreased depression in patients with CHF [10]. However, these studies are mostly focus on adults, while on whether psychotherapy benefits depression and cardiovascular outcome in the elderly patients with CHF are tiny.

Furthermore, psychological therapies in these studies are delivered by face-to-face interviews. A large proportion of CHF patients with depression may not get access to face-to-face psychological treatments due to various reasons, such as functional limitations in mobility, transportation problems, stigma concerns, time constraints or living in a rural area that lacks adequate mental health services, which restrict the use of these treatments [11, 12]. Delivered by telephone may not only overcome the barriers mentioned above but reduce attrition [13], with equivalent effective on depression [13, 14]. And most elderly patients prefer psychotherapy to medication [15]. Therefore, the telephone-delivered psycho-behavioural intervention may be a preferable choice for CHF patients with depression. However, effectiveness of telephone-delivered psycho-behavioural intervention for depression in CHF has not been established.

We design this study to examine the effects of the intensive and maintenance phase of a telephone-delivered psycho-behavioural intervention for depression among the elderly with CHF. Meanwhile, the study will explore the potential benefits for cardiovascular function and quality of life of the intervention. This paper describes the rationale and the design of the trial.

## Methods

The present study procedures and informed consent form, which conform with the principles outlined in the Declaration of Helsinki [16] for experiments involving humans, were approved by Ethics Board of all participating sites. The first patient was enrolled on 3 August and all study sites are still recruiting the subjects. Moreover, the trial was registered at www.clinicaltrials.gov (identification number: NCT03233451) on 28 July 2017 and updated on 18 August 2017.

### Design

**Fig. 1:**
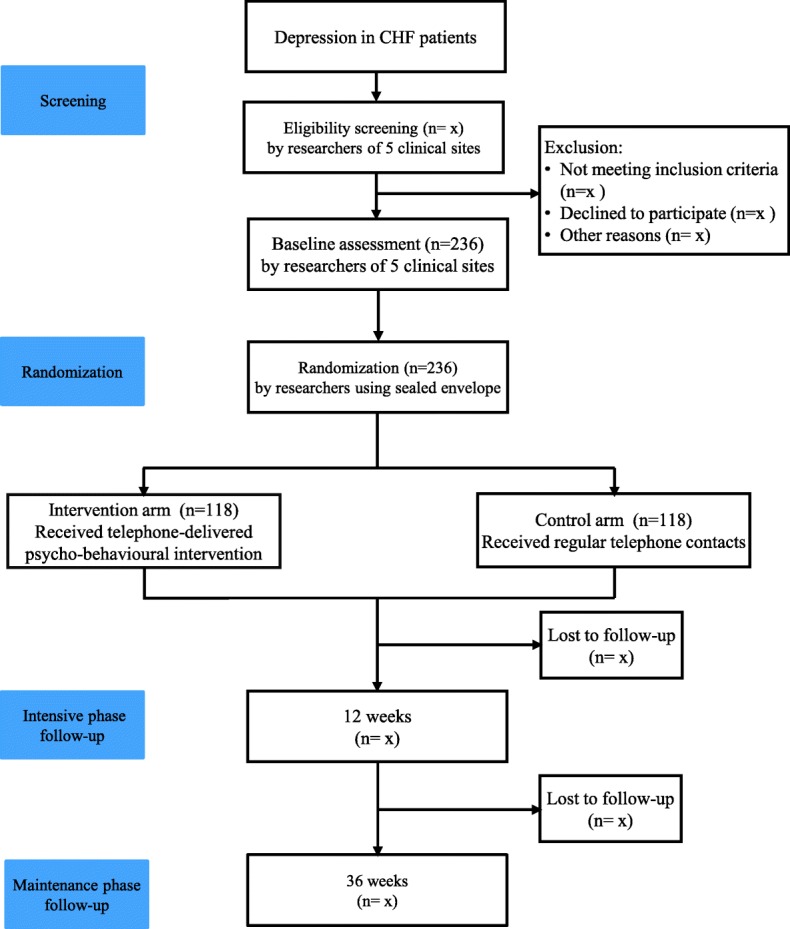
Flowchart of a telephone-delivered psycho-behavioural intervention on depression in the elderly with CHF

The study is designed as a 36-week prospective, multicentre, parallel, randomized, controlled trial (RCT) examining the effects of a telephone-delivered psycho-behavioural intervention. The study consists of two arms: a telephone-delivered psycho-behavioural intervention arm, and a control arm who will receive regular telephone contacts during the research period. Figure 1 shows the study process. This protocol is reported as according to the ‘Standard Protocol Items: Recommendations for Interventional Trials’ statement [17].

### Coordination and study sites

The trial team consists of a coordination management centre, five clinical sites (three medical centres affiliated with medical universities, one study sites secondary hospital and one community health centre), one mental health centre located in Beijing, China.

Funded by the Beijing Municipal Health Commission, Peking University Institute of Mental Health acts as the coordination management centre, and is responsible for designing the protocol, developing the manuals for telephone-delivered psycho-behavioural intervention and working procedure, coordinating the study sites, training all investigators and psychological counsellors, monitoring data collection, and managing the research funds.

Two hundred and thirty-six participants will be enrolled in five clinical sites. Based on the size of subject pool of clinical sites, the number of enrolments is assigned as follows: 59 patients will be respectively enrolled in three medical centres, 35 in one secondary hospital and 24 in one community health centre. Enrolments were started in 3 August 2017 and are expected to complete in August 2019. The statistical analysis will be completed by November 2019. The primary results are expected to be published by mid-2021.

### Participants

Approximately 236 elderly inpatients and outpatients with New York Heart Association (NYHA) classes II to III CHF are being recruited from those five clinical sites. Subjects are being recruited by the following two ways: (1) Using paper posters and advertisement through the WeChat Message Board managed by the coordination management centre. (2) Participants, who are interested in this project, will go to any of those five clinical sites to complete a screening questionnaire. (3) The cardiologists will refer outpatient or inpatient patients with CHF to the study coordinator in each clinical sites. A designated investigator of each study site will contact the potential eligibility participants and obtain informed consent for initial screening.

### Inclusion criteria

(1) Age between 60 and 85 years old; (2) Male or female; (3) Diagnosis of depression as measured with Patient Health Questionnaire 9 (PHQ-9) [18] score equal to or greater than 5 point; (4) Diagnosis with CHF according to the diagnostic criteria for CHF presented in the China Heart Failure Diagnosis and Treatment Guidelines [19], with NYHA grade II-III; (5) Reading and writing competency for completing the rating scales; (6) Sufficient physical condition, hearing and vision to ensure the completion of psycho-behavioural intervention.

### Exclusion criteria

(1) With a high risk of suicide, suicide attempts and suicidal behaviour, i.e., 17-item Hamilton Rating Scale for Depression (HAMD-17) [20] scores of 30 or above, HAMD-17 suicide subscale scores of 3 or above; or Mini-International Neuropsychiatric Interview (MINI) [21] scores of 6 or above; (2) With intact cognitive function and a Community Screening Instrument for Dementia (CSI-D) [22] score of less than 7; (3) Not receiving optimal and stable depression regimens; (4) Lifetime or current diagnosis of other psychotic disorder, cognitive impairment, schizophrenia, schizoaffective psychiatric disorders, delusional disorders, undefined psychotic disorders, substance and alcohol abuse; (5) Planned heart surgery within 9 months.

### Withdrawal criteria

Participants are free to withdraw from the study at any time without any influence on future treatment. (1) The participants readmit to hospital due to aggravation of heart failure. (2) Signs or symptoms of rapidly progressing diseases that impede assessment and treatment of this research. (3) It will cause damage to participants’ health if the participants continue to take part in the study. (4) Participants withdraw informed consent.

For every participant who withdraws from the study, the follow-up assessment will be collected for early termination. And the reasons for withdrawal will be recorded.

### Study interventions

Interventions will be delivered by medical care staffs all of whom had obtained the qualification of psychological counsellors for at least 1 year. All psychological counsellors will have received rigorous face-to-face training from an investigator from coordination management centre. Prior to the training, all psychological counsellors will receive a self-compiling manual for telephone-delivered psycho-behavioural intervention and a working manual for the trial. Self-compiling intervention manual outlines the protocol for treatment and is specifically targeted at treating depression among the elderly with CHF.

The trained psychological counsellors will provide manualized telephone-delivered psycho-behavioural intervention guidance to the intervention arm or regular telephone contacts to the control arm. All participants will receive the same usual clinical care from cardiologists, either in intervention or control arm.

### Intervention

The treatment of psycho-behavioural intervention aims to help patients to recognise depression and CHF related distress, identify unhealthy sleep patterns and unhelpful beliefs, increase physical activity, enhance coping skills, and change their daily unhealthy behaviour and negative cognition in order to enable adaptive ways of viewing specific situations or events.

The telephone-delivered psycho-behavioural intervention includes a 12-week intensive phase and a 24-week maintenance phase. All sections, according to a semi-structured psycho-behavioural intervention manual developed by coordination management centre, were specifically targeted at treating the depression in the elderly with CHF.

During the intensive phase, participants will receive eight weekly psycho-behavioural intervention sessions and four weekly subsequent assignments. During the eight weekly interventions, the psychological counsellor will deliver the intervention by telephone according to the self-compiling intervention manual. Each session lasts approximately 45–50 min, consisting of the introduction, main thematic conversation, and conclusion of the intervention. The themes of eight sessions were, in order, *“Introduction to depression”, “Sleep optimization”, “Change behaviour and cognition”, “Identifying negative thinking”, “Optimal cognition”, “Coordination of cognition and behaviour”, “Stress management”, and “Review and outlook”.* After 8 sessions of intervention, the psychological counsellor will give the subject assignments according to self-compiling manual for 4 weeks.

During 24-week maintenance phase the subject will receive monthly psycho-behavioural support provided by psychological counsellors, including a 10-min interview delivered by telephone.

Before the intervention, the participant will receive a manual to help them follow the guidance of the counsellors during the intervention. The main content of the manual includes an introduction to the intervention of each session, records template of behavioural activity and thought, monitoring sleep, self-assessments about pain and depression, and summaries.

### Control

The participants in the control arm will receive telephone-delivered regular telephone contacts from the trained psychological counsellors weekly. The regular telephone contacts include a 5–10 min’ telephone interview that does not include specific psychological intervention and assignments during intensive phase. During the maintenance phase, the subjects in the control group will receive monthly regular telephone contacts provided by psychological counsellors, including a 10-min interview by telephone.

### Sample size calculation

In the present study, the response rate of depression measured by QIDS-SR_16_ from baseline to 12 weeks was selected as the primary outcome. According to the result of a previous study [23], we assumed that the response rate of depression in the intervention and control arms will be 50% and 30% respectively, as we assumed that the intervention arm will be superior to the control arm in terms of depression improvement. With the type I error level at 5% and the power at 90%, the ratio of the two arms as 1:1, we estimate that 101 participants will be needed in each arm. With the assumption of a 15% loss to follow-up rate, each arm needed to recruit 118 participants, and therefore a total of 236 subjects would be randomised.

### Randomization and blinding

An independent biostatistician, from coordination management centre, created the randomisation, using the Microsoft Excel (Versions for Microsoft Windows 2016 Excel 16.0) RAND function in advance. To make sure the number of patients balanced in two arms, we use permuted block randomisation with variable block sizes of 4, 8 (randomly arranged). Allocations are concealed in sequentially numbered opaque envelopes and opened by the independent study coordinator among the study site after the baseline evaluation. After the baseline assessment, subjects will be randomly assigned in a 1:1 ratio at the site level to either the intervention arm or the control arm.

It is impossible for study coordinator, participants and counsellors to be blinded in this trial. However, other study personnel involved in recruitment, screening, data collection, entry, and analysis will be blinded to group assignment. Study coordinator, participants and counsellors are asked not to discuss sequence with study personnel blinded. The allocation for a specific participant will not be broken unless there is any suicidal risks and incidents occurs assessed by assessors or counsellors. Then this participant will be encouraged to seek help from the clinical site. And appropriate referral information will be provided to the cardiologist.

### Outcomes measurement

The primary outcome measure is the response rate of depression from baseline to week 12. The response rate was defined as a 50% or greater score reduction on Quick Inventory of Depressive Symptomatology-Self-Report (QIDS-SR_16_) [24].

The secondary outcome measures include the change in cardiac function, quality of life and severity of depressive symptoms during the intensive phase and the maintenance phase, respectively. The cardiac function as measured by the left ventricular ejection fraction via echocardiography and the plasma concentration of N-terminal pro-brain natriuretic peptide (NT-proBNP). The quality of life was measured by Minnesota Living with Heart Failure Questionnaire (MLHFQ) [25, 26]. The severity of depressive symptoms was measured the QIDS-SR_16_, HAMD-17, the Beck Depression Inventory II (BDI-II) [27] and Geriatric Depression Inventory-Self-Report (GDI-SR) [28].

### Assessment and follow-up

Screening assessment includes demographic information, physical examination (symptoms and signs of CHF), cardiac function with NYHA class, vital signs, past medical history, smoking history, depression symptomatology (PHQ-9 and HAMD-17) and other potential exclusion criteria. Those who met eligibility criteria will be invited to participate in trial.

Baseline assessment includes depression symptom QIDS-SR_16_, BDI-II, GDI-SR, MLHFQ, cardiac function (class of NYHA, 6-min walk distance, echocardiography, plasma concentration of NT-proBNP). NT-proBNP will be analysed at the central laboratory (Peking University Institute of Mental Health).

At follow up, participants will complete questionnaire same to that at baseline.

The participants will be evaluated at baseline, weeks 1, 2, 4, 8, 12, 24 and 36 with a set of questionnaires (see Table 1).

**Table 1:** Diagram of assessments

Measure	Screening	Baseline	Week 1	Week 2	Week 4	Week 8	Week 12	Week 24	Week 36	Early withdrawal
Depression and quality of life										
PHQ-9	X									
Suicide subscale of MINI	X									
CSI-D	X									
HAMD-17	X	X			X	X	X	X	X	X
QIDS-SR_16_		X	X	X	X	X	X	X	X	X
BDI-II		X	X	X	X	X	X	X	X	X
GDI-SR		X			X	X	X	X	X	X
MLHFQ		X			X	X	X	X	X	X
Cardiac function										
NYHA staging	X	X			X	X	X	X	X	X
6-min walk test	X	X			X	X	X	X	X	X
NT-proBNP		X					X		X	X
Ultrasound cardiogram		X					X		X	X
Clinical records and logs										
Clinical symptoms and signs	X	X			X	X	X	X	X	X
Vital signs	X	X			X	X	X	X	X	X
Concomitant medications		X			X	X	X	X	X	X
Adverse events log			X	X	X	X	X	X	X	X

*NYHA* New York Heart Association, *PHQ-9* Patient Health Questionnaire-9, *CSI-D* Community Screening Instrument for Dementia, *HAMD-17* Hamilton Rating Scale for Depression, *NT-proBNP* N-terminal pro-brain natriuretic peptide, *MLHFQ* Minnesota Living with Heart Failure Questionnaire, *BDI-II* Beck Depression Inventory-II, *QIDS-SR*_*16*_ Quick Inventory of Depressive Symptomatology–Self-Report, *GDI-SR* Geriatric Depression Inventory, self-report version

At 1- and 2-week follow-up, participants will receive a telephone call in which they will be administered the QIDS-SR_16_ and BDI-II. And then follow-up assessments will be conducted in the face-to-face follow-up clinic visits.

### Adherence

The definition of adherence to intervention is that all subjects must complete at least 2 semi-structured psycho-behavioural intervention sessions. There will be 5 times follow-up assessments conducted by face-to-face during clinic visits. The purpose of this assessment is to reduce the process of clinical visiting and reinforce adherence to treatment. Counsellors complete records on Case Report Form (CRF) after each session to identify themes or areas covered according to the intervention manual as a strategy to record adherence with the treatment.

### Quality control

An independent Data Safety and Monitoring Board (DSMB) is composed of three independent leaders with recognized clinical and research expertise in psychiatry, cardiology, and statistics. Every 6 monthly intervals, the DSMB will review the course of the trial, evaluate the safety data collected and monitor any unexpected events to ensure that the participants are not compromised. In addition, the DSMB can be approached on as needed. According to safety concerns, the committee of DSMB may, at any time, make recommendations to continue, modify or terminate the study.

The working manual was developed by coordination management centre, and finalized on 9 June, 2017. It outlines the protocol and the trial workflow for all investigators and psychological counsellors. It has been provided to each site to guide research relevant protocol and procedure, including screening, enrolment, assessment, echocardiogram data collection, data entering and checking and blood sample collection, storage and transportation.

In order to establish quality assurance, all the investigators and assessors will receive 2 days full and strictly training from coordination management centre according to the working manual. If investigators and assessors understand all requirements and their regulatory responsibilities in the study, they can participate in it. When a new staff joins in, he/she will be trained by an investigator from coordination management centre. In addition, a group of clinical experts, who are in cardiology and psychiatry, designated by the coordination management centre will gather monthly to review the protocol implementation and evaluate the reliability of clinical assessment.

A self-compiling manual for psycho-behavioural intervention was developed by Peking University Institute of Mental Health. It outlines the protocol for treatment and be specifically targeted at treating depression among the elderly with CHF. All psychological counsellors will receive strictly training about the working manual and telephone-delivered psycho-behavioural therapy before treatment start. And an experienced psychological counsellor from Peking University Shougang Hospital will monthly supervise those trained counsellors.

Each site, responsible for recruiting, screening, enrolling and assessing participants, has a principal investigator (responsible for all work within the site), one study coordinator (responsible for the coordination in this site, supervising compliance to study protocol, monitoring the progress of the study and controlling research quality), 1 to 2 cardiologists (responsible for recruitment, heart function evaluation and echocardiogram data collection), 2 to 4 assessors (responsible for recruitment and assessment), a nurse (responsible for blood sample collection and transportation), a data typist (responsible for entering paper-based questionnaire data into a web-based data system in time).

The investigators of coordination management centre will visit the sites weekly. And feedbacks on the course, main problems and recommendation will be notified to all investigators. All principal investigators and study coordinator will be called for a face-to-face meeting to discuss the difficulties and share experience each quarter to ensure the study quality.

### Data management

The coordination management centre developed the paper-based questionnaire and web-based data system for data collection checking and storage. All assessor, typist and cardiologists involved in the research will be given an ID number and password respectively by study coordinator of study sites. When completing each assessment, the assessor will record the data on paper-based questionnaire. After the hard copy of the questionnaire is checked by the study coordinator, the typist will input the data into the web-based data system in time. In order to protect the privacy of the participants, the web-based data system uses unique codes to represent the corresponding subject’s demographic information. The study coordinators from coordination management centre is authorized to review the study data. If there are queries, they will provide feedback to the assessor or clinician through online communication system. The database related to the trial will be securely retained by each site and will be kept for 5 years beyond trial completion. Per requirement by the Ethics Board, only authorized project staff has right to access to the database.

### Statistical analysis plan

For the demographic and other baseline characteristics of the subjects, summary statistics will be conducted, including mean (standard deviation) or median (25th -75th percentile) for continuous variables and frequency (percentage) for categorical variables. The patient characteristics will be compared using Chi-square test for categorical variables and Student’s t test for continuous variables, as appropriate.

For the primary outcome, Logistic regression model adjusting for clinical sites will be used to examine the difference in the response rate of depression at the end of intensive phase between the two arms. For the secondary outcomes, mixed-effects linear regression models will be used to test the change of the difference in cardiac function, quality of life and severity of depressive symptoms over the intensive phase and the maintenance phase, respectively, with the fixed effect of treatment, time, clinical sites, the interaction of treatment and time, as well as the random effect of patient.

The primary analysis of the study will be conducted based on the intention-to-treat principle (ITT), using data for all subjects who are randomized. Missing data will be replaced with the last assessments in order to achieve complete datasets. A per-protocol analysis will also be performed on the difference of the response rate of depression at the end of acute phase between the two arms, using data for those participants who completed 12 weeks of treatment and QIDS-SR_16_ both at baseline and the end of intensive phase.

All statistical analyses will be performed using statistical package for the social sciences (SPSS) software version 23.0. All *P* values will be two-sided. A *P* value of less than 0.05 will be considered to be statistically significant.

## Discussion

With this prospective, multicentre, parallel, randomized controlled trial, we aim at investigating the effect of a telephone-delivered psycho-behavioural intervention intended to improve the depression and cardiac function and quality of life of the elderly suffering from CHF with depression. To our knowledge, this study is the first prospective randomized controlled trial to assess the effect of a telephone-delivered psycho-behavioural intervention on the depression and cardiac function of the elderly suffering from CHF with depression in China.

This trial has several advantages. Firstly, this is, to our knowledge, the first trial about integration of psychological counsellors and cardiologists in China. It is multidisciplinary cooperation to explore treatments for depression in elderly patients with CHF. Secondly, the present study design uses telephone to deliver psycho-behavioural intervention for depression in the elderly patients with CHF. The telephone-delivered nature of the psycho-behavioural intervention will allow easy access regardless of the mobility, time constraints and transportation challenges of the elderly participants [11]. The treatment sections are scheduled according to the subjects’ preference; such flexibility promotes the retention of subjects in the study [13]. Thirdly, study participants are recruited from three medical centres affiliated with universities, one secondary hospital and one community health centre. The study participants are representative. And the multi-centre nature of the trial could enhance the opportunity to generalize of the findings. Fourthly, the study has little bias due to the design of prospective, randomized, controlled trial. At last, the present trial not only aims to test effects of a telephone-delivered psycho-behavioural intervention for depression among the elderly with CHF but also explores the potential benefits for cardiovascular function and quality of life during the intervention.

The challenge and limitation of this study is that we cannot perform double-blind plan. We must give the eligible participants sufficient information of the study protocol before randomising. In order to provide telephone-delivered psycho-behavioural intervention to the intervention arm or regular telephone contacts to the control arm, the psychological counsellors must be clearly told which arm the eligible participants were randomly assigned to, according to the protocol. However, all researchers, excluding study coordinators and psychological counsellors, are blinded to the group assignments. To some extent, it may be difficult to invite patients with CHF to take part in the study. There may be ignorance of depression in the elderly CHF [5]. The symptoms of depression, such as feeling of fatigue, low energy, sleep disorder and weight loss or gain, are represented by the similarities with as symptoms of CHF [29]. The elderly participants with CHF may refuse to accept psycho-behavioural intervention due to above reasons. However, we regularly go to the study sites to give lectures of depression with CHF for potential participants. We draw the potential participants interesting through the WeChat and posters. We give the participants, who join the trial, and their family cares health education and supervision management. And we ensure that all potential eligible participants are fully informed about the content of trial before getting consent. At the same time, the study staff will make every effort to maintain keep contact with the participants to secure a reasonable retention rate.

## Conclusion

In conclusion, if the study shows that the telephone-delivered psycho-behavioural intervention is effective, the intervention may be a viable alternative for older adults with depression, particularly for those who prefer non-pharmacological and face-to face psychological interventions. Telephone-delivered psycho-behavioural intervention is feasible for patients with limited mobility. At last, the patients would receive evidence-based psychological intervention and might gain further benefits in terms of cardiac function.

## Abbreviations

BDI-II: Beck Depression Inventory II
CBT: Cognitive behaviour therapy
CHF: Chronic heart failure
CRF: Case Report Form
CSI-D: Community Screening Instrument for Dementia
DSMB: Data Safety and Monitoring Board
GDI-SR: Geriatric Depression Inventory-Self-Report
HAMD-17: 17-item Hamilton Rating Scale for Depression
ITT: Intention-to-treat principle
MINI: Mini-International Neuropsychiatric Interview
MLHFQ: Minnesota Living with Heart Failure Questionnaire
NT-proBNP: N-terminal pro-brain natriuretic peptide
NYHA: New York Heart Association
PHQ-9: Patient Health Questionnaire 9
QIDS-SR_16_: Quick Inventory of Depressive Symptomatology-Self-Report
RCT: Randomized, controlled trial
SADHART-CHF: Safety and Efficacy of Sertraline for Depression in Patients with CHF
SPSS: Statistical Package for the Social Sciences
